# Cysteine residues exposed on protein surfaces are the dominant intramitochondrial thiol and may protect against oxidative damage

**DOI:** 10.1111/j.1742-4658.2010.07576.x

**Published:** 2010-03

**Authors:** Raquel Requejo, Thomas R Hurd, Nikola J Costa, Michael P Murphy

**Affiliations:** MRC Mitochondrial Biology Unit, Wellcome Trust/MRC BuildingCambridge, UK

**Keywords:** cysteine, glutathione, mitochondria, peroxynitrite, protein thiol

## Abstract

Cysteine plays a number of important roles in protecting the cell from oxidative damage through its thiol functional group. These defensive functions are generally considered to be carried out by the low molecular weight thiol glutathione and by cysteine residues in the active sites of proteins such as thioredoxin and peroxiredoxin. In addition, there are thiols exposed on protein surfaces that are not directly involved with protein function, although they can interact with the intracellular environment. In the present study, in subcellular fractions prepared from rat liver or heart, we show that the quantitatively dominant free thiols are those of cysteine residues exposed on protein surfaces and not those carried by glutathione. Within the mitochondrial matrix, the concentration of exposed protein thiols is 60–90 mm, which is approximately 26-fold higher than the glutathione concentration in that compartment. This suggests that exposed protein thiols are of greater importance than glutathione for nonenzyme catalysed reactions of thiols with reactive oxygen and nitrogen species and with electrophiles within the cell. One such antioxidant role for exposed protein thiols may be to prevent protein oxidative damage. In the present study, in mitochondrial membranes and in complex I, we show that exposed protein thiols protect against tyrosine nitration and protein dysfunction caused by peroxynitrite. Therefore, exposed protein thiols are the dominant free thiol within the cell and may play a critical role in intracellular antioxidant defences against oxidative damage.

## Introduction

The thiol functional group plays a major role in intracellular antioxidant defences. Cysteine residues in the active sites of proteins such as thioredoxin (Trx), glutaredoxin (Grx) and peroxiredoxin (Prx) detoxify reactive oxygen species (ROS) and reactive nitrogen species and reduce oxidized protein thiols [1,2]. The low molecular weight thiol glutathione (GSH) acts in conjunction with GSH peroxidases, Grxs and glutathione *S*-transferases to detoxify ROS and electrophiles and to recycle oxidized protein thiols [3]. In addition to these enzyme-catalysed reactions, thiols can also react directly with some ROS and reactive nitrogen species; therefore, solvent-exposed thiols within cells may contribute to endogenous antioxidant defences [1,4,5]. Consequently, cysteine residues exposed on the surface of proteins without a clear functional or structural role may still make an important contribution to antioxidant defences [2]. However, this possibility is not widely recognized and there is little experimental evidence to support a protective role for exposed protein thiols. One factor impeding progress is the assumption that GSH is the quantitatively dominant intracellular thiol. Although a number of studies have investigated the intracellular abundance of protein thiols [2,5–8], little is known about the amount of exposed protein thiols within cells in comparison to GSH, or whether they are important in cellular defence. To determine the contribution of exposed protein thiols to the intracellular redox environment, we have measured their abundance on native proteins from tissue subfractions relative to the amount of GSH, quantified exposed protein thiols within isolated mitochondria and determined whether these protein thiols can protect against oxidative damage caused by peroxynitrite (ONOO^−^). These findings indicate that the cysteine residues exposed on the surface of proteins are the dominant intracellular thiol and that they may play an important role in intracellular antioxidant defences.

## Results

### Quantification of exposed protein thiols and GSH in tissue subfractions

**Fig. 1:**
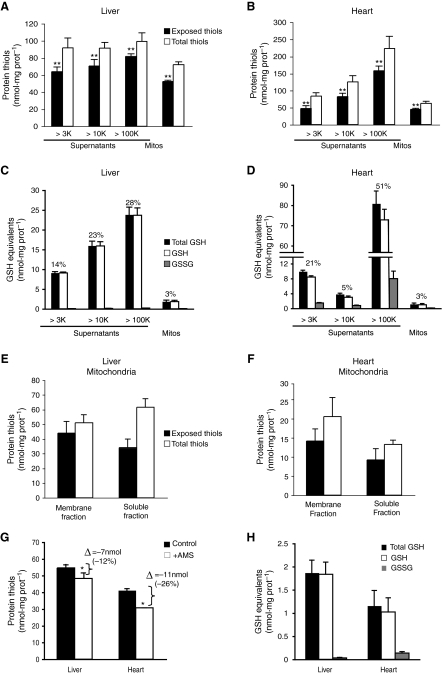
Total and exposed protein thiols and GSH in liver and heart tissue homogenates and mitochondria. (A, B) Total and exposed protein thiols in sequential supernatants from 3000 ***g***, 10 000 ***g*** and 100 000 ***g*** centrifugations, and from a mitochondrial fraction, isolated from liver (A) and heart (B) tissue homogenates. **P < 0.01 for comparison of total and exposed thiols by Student’s *t*-test. (C, D) Total GSH equivalents, GSH and 2× GSSG, in sequential supernatants from 3000 ***g***, 10 000 ***g*** and 100 000 ***g*** centrifugations, and from a mitochondrial fraction, isolated from liver (C) and heart (D) tissue homogenates. The percentages above the data bars indicate the total GSH content of the fraction as a percentage of its exposed protein thiol content. (E, F) Total and exposed thiols in membrane and matrix fractions from liver (E) or heart (F) mitochondria. Mitochondria (5 mg·mL^−1^ protein) were suspended in KCl buffer, pelleted by centrifugation and separated into membrane and matrix fractions and then exposed and total protein thiols were measured. (G) Exposed mitochondrial protein thiols ± the thiol alkylating agent AMS. Mitochondria (5 mg·mL^−1^ protein) were incubated in KCl buffer ± AMS (100 μm) for 10 min at 30 °C. Samples were then centrifuged and exposed protein thiols were measured. (H) GSH content of rat liver and heart mitochondria. Mitochondria (5 mg·mL^−1^ protein) were incubated in KCl buffer for 10 min at 30 °C and the GSH and GSSG contents measured. All data are the mean ± SD of three independent experiments.

To assess the importance for antioxidant defence of exposed thiols on the surfaces of proteins in their native conformations, we quantified exposed and total protein thiols in tissue subfractions (Fig. 1). Tissue homogenates from rat liver and heart were fractionated by sequential differential centrifugation to give supernatants from the 3000 ***g*** (crude homogenate), 10 000 ***g*** (cytosol and microsomes) and 100 000 ***g*** centrifugations (soluble cytosol fraction) and a mitochondrial fraction (pellet from the 10 000 ***g*** centrifugation). To measure exposed protein thiols, we used the mild detergent *n*-dodecyl-β-d-maltopyranoside (DDM) to solubilize membrane proteins with minimal disruption to protein conformation. The suspensions were then treated with dithiothreitol to reduce thiols that had become reversibly oxidized during fractionation. The dithiothreitol and low molecular weight thiols such as GSH were then removed by centrifugal gel filtration and exposed protein thiols were measured using 5,5′-dithiobis(2-nitrobenzoic acid) (DTNB). Control experiments showed that lysing mitochondria by freeze/thawing instead of with DDM treatment gave similar levels of exposed thiols (data not shown). Total protein thiols were measured after complete denaturation of the proteins with SDS. Exposed and total protein thiols for each fraction are shown in Fig. 1A,B, for liver and heart, respectively. The total protein thiols in the fractions were in the range 50–225 nmol·mg protein^−1^. Allowing for variation in cysteine content between different tissues and subcellular fractions, these values are consistent with the known cysteine content of mammalian proteins of approximately 2% of amino acid residues. On average, approximately 70% of total protein thiols were exposed to the solvent (range 56–84%).

We next measured GSH and glutathione disulfide (GSSG) in each fraction prior to dithiothreitol treatment or centrifugal filtration (Fig. 1C, D). Most of the GSH pool was present as GSH and the total GSH content varied in the range 2–80 nmol·mg protein^−1^ (Fig. 1C, D). The total amounts of GSH equivalents in each fraction as a percentage of exposed protein thiols are also shown above the data bars (Fig. 1C, D). In all fractions, the GSH content was substantially less that that of exposed protein thiols, in the range 3–51%. Because GSH is by far the most abundant intracellular low molecular thiol, this demonstrates that exposed protein thiols are the quantitatively dominant intracellular thiol and, in some cases, are present at a 20–30-fold higher concentration than GSH. This finding is consistent with exposed protein thiols playing a role in intracellular antioxidant defences.

### Quantification of exposed protein thiols and GSH within mitochondria

To further analyse the potential role of surface protein thiols in antioxidant defences, we next focussed on their role within mitochondria. This was carried out because: mitochondria are a major source of ROS within the cell [9] and, consequently, have extensive antioxidant defences; the pH in the mitochondrial matrix (∼ 7.8) is higher than in the cytosol (7.2), rendering protein thiols (typical p*K*_a_∼ 8–9) more reactive for processes requiring the thiolate; and, finally, mitochondria have experimental advantages because they are discrete, closed systems with their own GSH, Trx, thioredoxin reductase (TrxR), NADPH and Grx systems that can be investigated under conditions that are physiologically relevant.

First, we quantified exposed and total protein thiols in membrane and soluble fractions from liver and heart mitochondria (Fig. 1E, F). Approximately 70% of total protein thiols were exposed to the solvent (range 55–85%) (Fig. 1E, F). However, these measurements cannot distinguish exposed protein thiols on the mitochondrial outer membrane, the intermembrane space and on the outer face of the inner membrane from those within the mitochondrial matrix. Because matrix protein thiols are of the greatest interest as a result of the elevated oxidative stress of that compartment, we measured these by blocking nonmatrix protein thiols with the membrane impermeant thiol alkylating agent 4-acetamido-4′-maleimidylstilbene-2,2′-disulfonic acid (AMS) (Fig. 1G). AMS decreased the total amount of exposed protein thiols by 7 nmol·mg protein^−1^ (−12%) in liver mitochondria and by 11 nmol·mg protein^−1^ (−26%) in heart mitochondria (Fig. 1G). Thus, the amount of exposed protein thiols is approximately 48 and 31 nmol·mg protein^−1^ within the matrices of liver and heart mitochondria, respectively. This is 25–30-fold higher than their GSH contents of 1–2 nmol·mg protein^−1^ (Fig. 1H). The mitochondrial matrix volume under these conditions is approximately 0.5 μl·mg protein^−1^ [10], giving a concentration of GSH of approximately 3 mm, which contrasts with the matrix concentration for exposed protein thiols of 60–90 mm. Therefore, within the mitochondrial matrix, exposed cysteine residues on the surface of proteins are by far the dominant free thiol.

### Response of exposed mitochondrial protein thiols to oxidative stress

**Fig. 2:**
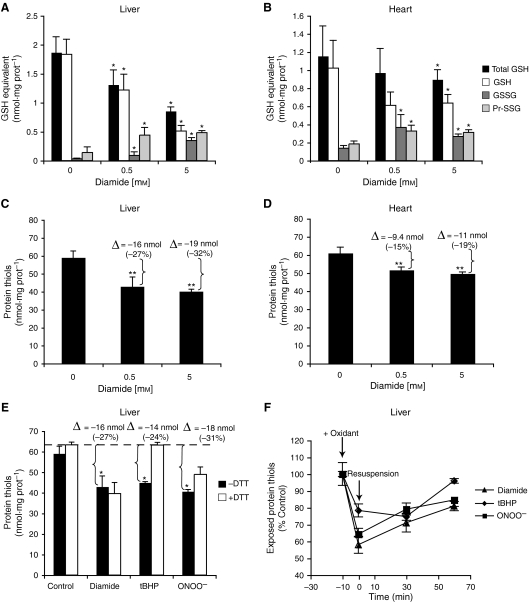
Exposed protein thiols and GSH in oxidatively stressed mitochondria. (A–D) Effect of diamide on exposed protein thiols, protein-GSH mixed disulfides and GSH. Mitochondria (5 mg·mL^−1^ protein) from the liver (A, C) or heart (B, D) were incubated with diamide for 5 min at 37 °C. The values after the Δ in (C) and (D) are the actual and the percentage changes in protein thiols relative to controls. (E) Effects of oxidants and dithiothreitol on exposed mitochondrial protein thiols. Liver mitochondria (5 mg·mL^−1^ protein) were incubated for 5 min with 0.5 mm ONOO^−^, tBHP or diamide and exposed protein thiols measured. For some incubations, the mitochondria were incubated with 1 mm dithiothreitol before measurement of protein thiols. The values after the Δ in (C) and (D) are the actual and the percentage changes in protein thiols relative to controls. (F) Reduction of mitochondrial thiols after oxidative stress. Liver mitochondria (5 mg·mL^−1^ protein) were incubated with either carrier, 0.5 mm tBHP, ONOO^−^ or diamide for 10 min. Next, mitochondria were pelleted by centrifugation and resuspended in medium without oxidant. The exposed protein thiols were measured as a percentage of parallel control incubations that had undergone the same isolation and resuspension procedures but without exposure to oxidant. All data are the mean ± SD of three experiments: *P < 0.05, **P < 0.01 relative to controls by Student’s *t*-test. DTT, dithiothreitol.

The high concentration of exposed protein thiols within the mitochondrial matrix is consistent with them playing a role in antioxidant defence. If this is the case, then their redox state should respond to mitochondrial oxidative stress. Treatment of liver or heart mitochondria with diamide oxidized the matrix GSH pool, decreased the GSH content by 1–1.5 nmol·mg protein^−1^ and led to the formation of GSSG and up to 0.4 nmol·mg protein^−1^ of protein mixed disulfides (Fig. 2A, B). Under these conditions, there was a loss of 9–19 nmol·mg protein^−1^ of exposed protein thiols, corresponding to 15–32% of the total present (Fig. 2C, D). Similarly, treatment of liver mitochondria with *tert*-butyl hydrogen peroxide (tBHP) or ONOO^−^ oxidized 14–18 nmol·mg protein^−1^ exposed protein thiols, corresponding to 24–31% of the total present (Fig. 2E). Oxidation of exposed protein thiols by tBHP was fully reversed by dithiothreitol, whereas that by ONOO^−^ was partially reversed and that by diamide was not reversed (Fig. 2E), presumably as a result of the formation of higher thiol oxidation states such as sulfinic and sulfonic acids that are not reduced by dithiothreitol [11]. When stressed mitochondria were washed to remove the oxidant and reincubated, the oxidation of exposed protein thiols was partially restored by intra-mitochondrial reduction processes (Fig. 2F). Therefore, during oxidative stress, the extent of thiol modification of exposed protein thiols is ten to 20-fold greater in magnitude than that of the entire GSH pool, and a proportion of the changes to exposed protein thiols can be reversed. These findings are consistent with exposed protein thiols within mitochondria playing an antioxidant role during their response to oxidative stress.

### Protection against ONOO^−^-induced tyrosine nitration by exposed protein thiols

The data shown in Figs 1 and 2 reveal that there is a high concentration of exposed protein thiols within mitochondria that respond to oxidative stress. To determine whether these exposed protein thiols could protect mitochondrial proteins against oxidative damage, we next investigated isolated mitochondrial membranes. This system contains an active respiratory chain and has a large number of exposed thiols that are easily accessible and measureable [12–14]. As an oxidant, we chose ONOO^−^ because it contributes to mitochondrial oxidative damage in a range of pathologies [15] and is known to react with protein thiols [16]. An important mode of damage caused by ONOO^−^ is the specific oxidation of protein tyrosine residues to 3-nitrotyrosine by a two step process involving the initial formation of a tyrosyl radical, which then goes on to react with a ^•^NO_2_ radical to form nitrotyrosine [15,17]. Because the formation of 3-nitrotyrosine can be measured using a specific antibody [17], the determination of the effect of exposed protein thiols on tyrosine nitration in mitochondrial membranes serves to indicate whether exposed protein thiols can be involved in antioxidant defences.

**Fig. 3:**
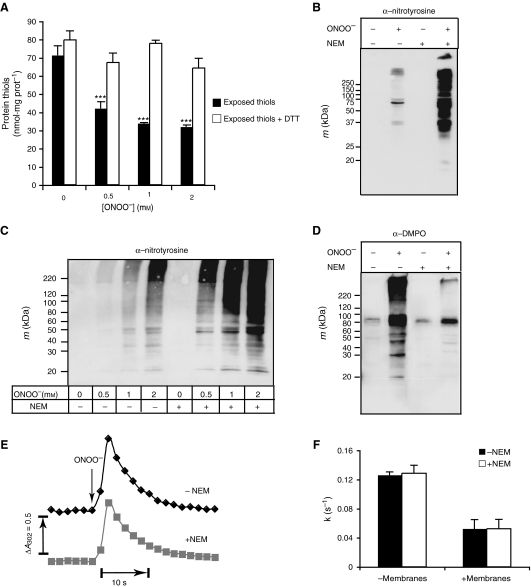
Effect of blocking exposed protein thiols with *N*-ethylmaleimide on nitration by ONOO^−^ of mitochondrial membrane proteins. (A–D) Mitochondrial membranes were incubated (20 mg·mL^−1^ protein) at 37 °C in membrane buffer with either no additions or after pre-treatment with 1 mm*N*-ethylmaleimide for 10 min. Next, the membranes were exposed to different doses of ONOO^−^, or decomposed ONOO^−^ for the control incubations, for 5 min. (A) After incubation exposed protein thiols were measured. Data are the mean ± SD of three experiments. ***P < 0.001 by Student’s *t*-test. (B) After incubation mitochondrial membranes (75 μg of protein) was separated by SDS-PAGE and immunblotted to detect 3-nitrotyrosine residues. (C) After incubation, mitochondrial membranes (50 μg of protein) was separated by SDS-PAGE and immunoblotted to detect 3-nitrotyrosine residues. (D) Membranes were incubated as above but in the presence of DMPO (100 mm). After incubation, mitochondrial membranes (75 μg of protein) was separated by SDS-PAGE and immunoblotted to detect DMPO protein adducts. (E, F) Rate of decay of ONOO^−^. (E) The decomposition of ONOO^−^ (1 mm) was monitored by measuring *A*_302_ after its addition to a rapidly stirred suspension of membranes (1 mg^·^mL^−1^ protein) incubated as described above in presence or absence of 1 mm*N*-ethylmaleimide. (F) Rate constants for decomposition of ONOO^−^ in controls or samples containing mitochondrial membranes, with or without *N*-ethylmaleimide. Data are the mean ± SD of three experiments. DTT, dithiothreitol; NEM, *N*-ethylmaleimide.

There were approximately 85 nmol·mg protein^−1^ total protein thiols in mitochondrial membranes and approximately 70 nmol·mg protein^−1^ of these were exposed to the solvent (Fig. 3A). There was a dose-dependent decrease in exposed protein thiols on reaction with ONOO^−^ that was largely reversed by dithiothreitol, consistent with the oxidation of protein thiols by ONOO^−^ to thiyl radicals and sulfenic acids [16] (Fig. 3A). The reaction of ONOO^−^ with mitochondrial membranes also formed 3-nitrotyrosine from tyrosine residues, as indicated by immunoblotting with a specific antibody (Fig. 3B). The formation of 3-nitrotyrosine was dependent on the concentration of ONOO^−^ (Fig. 3C). To determine whether exposed protein thiols decreased 3-nitrotyrosine formation, we pre-treated membranes with *N*-ethylmaleimide to block all exposed thiols. This rendered tyrosine residues in the membranes far more susceptible to nitration on exposure to ONOO^−^ (Fig. 3B, C)*.* To determine whether thiyl radicals were formed on the cysteine residues of membrane proteins during exposure to ONOO^−^, we added the spin trap 5,5-dimethyl-1-pyrroline-*N*-oxide (DMPO), which forms stable protein adducts with thiyl radicals that can be detected on immunoblots [18]. This experiment demonstrated the *N*-ethylmaleimide-sensitive formation of DMPO-protein adducts, which is consistent with protein thiol oxidation by ONOO^−^ (Fig. 3D).

The data shown in Fig. 3B, C indicate that blocking exposed protein thiols with *N*-ethylmaleimide renders membrane proteins more susceptible to nitration by ONOO^−^. We suggest that this occurs because *N*-ethylmaleimide blocks thiols, thereby preventing cysteine residues from protecting tyrosine residues from nitration. However, an alternative interpretation is that exposed protein thiols react rapidly with ONOO^−^ to accelerate its degradation, and that *N*-ethylmaleimide treatment may slow this process, thereby enhancing nitration by increasing the bulk exposure of tyrosines to ONOO^−^. To determine whether this could be the case, we investigated the effect of *N*-ethylmaleimide treatment on the rate of decay of ONOO^−^. Accordingly, ONOO^−^ was injected into a rapidly stirred membrane suspension ± *N*-ethylmaleimide and the absorption of ONOO^−^ was measured over time (Fig. 3E). The first-order decay process was analysed to generate rate constants for the decay of ONOO^−^ (Fig. 3F). In the absence of membranes, the ONOO^−^*t*_1/2_ was approximately 5 s and, in the presence of membranes, the *t*_1/2_ increased to approximately 13 s (Fig. 3F), probably as a result of permeation of ONOO^−^ into the hydrophobic membrane core [19]. In the presence or absence of membranes, the rate of decay of ONOO^−^ was unaffected by *N*-ethylmaleimide (Fig. 3E, F). Therefore, *N*-ethylmaleimide treatment does not alter membrane exposure to the bulk of the added ONOO^−^ and the increased membrane nitration in the presence of *N*-ethylmaleimide is a result of cysteine residues blocking tyrosine nitration by ONOO^−^ by local interactions and not a result of the effects on the overall concentration of ONOO^−^ added to the suspension.

### Exposed protein thiols protect complex I against damage by ONOO^−^

Having shown that exposed protein thiols decreased tyrosine nitration in mitochondrial membranes, we next investigated whether the prevention of nitration had functional consequences for the proteins affected. Accordingly, we investigated whether exposed protein thiols could protect mitochondrial complex I from ONOO^−^ damage. Complex I was chosen because it is a major component of the mitochondrial respiratory chain and is known to be readily nitrated and inactivated by ONOO^−^ both *in vitro* and *in vivo* [20–22]. Furthermore, complex I has a large number of redox-active exposed thiols on its surface that interact with the GSH pool and have been suggested to play a role in protecting the complex from oxidative damage [14,23].

**Fig. 4:**
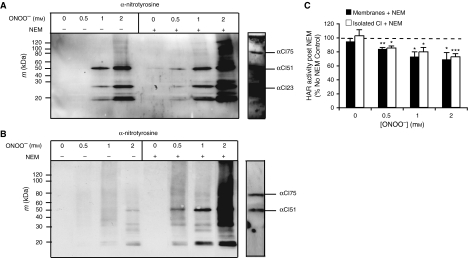
Nitration and inhibition of complex I by ONOO^−^. (A) Mitochondrial membranes were incubated as described in Fig. 3A–D with the indicated concentrations of ONOO^−^ or of decomposed ONOO^−^ for controls. Next, membrane samples (∼ 150 μg of protein per lane) were separated by BN-PAGE, the complex I band excised and further separated by SDS-PAGE and then immunoblotted using an antibody against 3-nitrotyrosine. The blot was reprobed using antisera against the 75 kDa, 51 kDa or 23 kDa complex I subunits and one lane of this is shown. (B) Isolated complex I (25 μg) was incubated in 50 μL of KCl buffer at 37 °C for 10 min ± *N*-ethylmaleimide, then the indicated concentrations of ONOO^−^, or decomposed ONOO^−^ for controls, were added and the samples were processed 5 min later. For this, 300 μL of ethanol was added and, after overnight incubation at −20 °C, protein was pelleted, suspended in loading buffer, and 10 μg of protein was separated by SDS-PAGE and immunblotted using an antibody against 3-nitrotyrosine. The blot was reprobed using antisera against the 75 kDa and 51 kDa complex I subunits and one lane of this is shown. (C) The activity of complex I measured as NADH:HAR oxidoreductase activity in membranes and isolated complex I. Data are the mean ± SD of three independent measurements and are the percentage of parallel control measurements. Data are the mean ± SD of three measurements (*P < 0.05, **P < 0.01, ***P < 0.001). NEM, *N*-ethylmaleimide.

First, the effects of ONOO^−^ on complex I nitration in mitochondrial membranes were examined (Fig. 4). Accordingly, we exposed membranes to ONOO^−^, then isolated complex I by blue native-PAGE (BN-PAGE) and further separated the complex into its constituent subunits by SDS-PAGE in the second dimension [23] (Fig. 4A). This process isolated complex I, as confirmed by re-probing the immunoblots for the complex I 75 kDa, 51 kDa and 23 kDa subunits (Fig. 4A). This process revealed that there was extensive nitration of complex I subunits in membranes exposed to ONOO^−^ and that this nitration was increased by *N*-ethylmaleimide pre-treatment (Fig. 4A). When isolated complex I was incubated with ONOO^−^, this also led to tyrosine nitration that was greatly enhanced by pre-treatment of complex I with *N*-ethylmaleimide (Fig. 4B).

To determine whether the increased nitration of complex I by ONOO^−^ in the presence of *N*-ethylmaleimide had any functional impact, we next assessed the effect of ONOO^−^ on complex I activity. Because alkylating complex I thiols with *N*-ethylmaleimide inhibits its NADH-ubiquinone oxidoreductase activity, we instead investigated the NADH-dependent reduction of hexa-ammineruthenium (III) chloride (HAR) by complex I [24]. This assay measures the activity of the flavin mononucleotide site of complex I and is not inhibited by *N*-ethylmaleimide. Furthermore, the flavin mononucleotide binding site is on the 51 kDa subunit of complex I, and the data in Fig. 4A,B suggest that this subunit is likely to be extensively nitrated by ONOO^−^ and that *N*-ethylmaleimide renders the 51 kDa subunit more susceptible to nitration. The HAR activity of complex I in membranes and in the isolated complex were measured in *N*-ethylmaleimide-treated membranes or isolated complex I as a percentage of the activities in non *N*-ethylmaleimide-treated controls (Fig. 4C). *N*-ethylmaleimide treatment did not affect the HAR activity of either the complex in membranes or of the isolated complex. However, in the presence of *N*-ethylmaleimide, ONOO^−^ inhibition of HAR activity was significantly enhanced (Fig. 4C). This is consistent with exposed thiols on complex I protecting it from oxidative damage and indicates that the inhibition of complex I HAR activity by ONOO^−^ correlates with the extent of tyrosine nitration.

### Recycling of oxidized protein thiols by GSH

The data obtained so far support a role for surface protein thiols in protecting protein tyrosine residues from nitration by ONOO^−^. However, through this reaction, an exposed protein thiol will be converted to a sulfenic acid or a thiyl radical [16], which may react with O_2_ to become irreversibly oxidized to a sulfinic or sulfonic acid, damaging the protein and preventing the thiol from protecting tyrosine residues any further. For exposed cysteine residues to be effective antioxidants, it is important for the sulfenic acid or thiyl radical to be rapidly recycled back to a thiol. One way in which this may occur is by the sulfenic acid/thiyl radical reacting with GSH to generate a mixed disulfide, or a radical anion mixed disulfide, respectively. The radical anion mixed disulfide would then lose its electron to O_2_ by the Winterbourn reaction [25]. These reactions would convert the partially oxidized thiols to protein GSH mixed disulfides, which are rapidly reduced to a thiol by the GSH pool and Grx in mitochondria and on complex I [13,14].

**Fig. 5:**
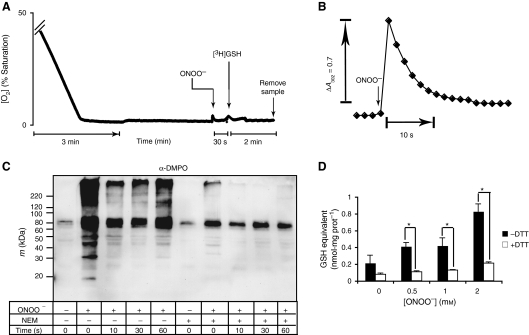
Glutathionylation of exposed protein thiyl radicals. Mitochondrial membranes (1 mg·mL^−1^ protein) were incubated in 1 mL of membrane buffer containing 2 mm succinate and 4 μg of rotenone within a rapidly stirred O_2_ electrode chamber. (A) Anaerobic incubation of mitochondrial membranes. An O_2_ electrode trace of a typical mitochondrial membrane experiment is shown. Respiration by the membranes consumes all of the O_2_, causing the incubation to become anaerobic. The points corresponding to those at which ONOO^−^ and [^3^H]GSH were added and where the samples were taken for analysis for the experiment in (D) are indicated on the trace. (B) Time course of ONOO^−^ decay. Mitochondrial membranes (1 mg·mL^−1^ protein) were stirred rapidly in a 3 mL sealed, anaerobic cuvette and, where indicated, 1 mm ONOO^−^ was added and its decay followed at 302 nm. (C) Stability of protein radicals produced by the treatment with ONOO^−^. Mitochondrial membranes were incubated at room temperature ± *N*-ethylmaleimide (NEM) (1 mm) for 10 min under anaerobic conditions. Next, ONOO^−^ (0.5 mm) was added at various times, and then DMPO (100 mm) was added. The membrane proteins (150 μg of protein) were separated by SDS-PAGE and immunoblotted to detect DMPO-protein adducts. (D) Mitochondrial membranes were incubated anaerobically as above. Next, the indicated concentrations of ONOO^−^ were added, 30 s later 100 μm [^3^H]GSH was added and, 2 min later, membranes were isolated, treated ± dithithreitol and the content of [^3^H]GSH determined by scintillation counting. Data are the mean ± SD of three experiments. *P < 0.05 by Student’s *t*-test. DTT, dithiothreitol.

To determine whether this recycling pathway is possible, we investigated the reaction of GSH with partially oxidized protein thiols in our system. In doing so, we could not add GSH and ONOO^−^ to mitochondrial membranes at the same time because it would not be possible to distinguish the reaction of a protein sulfenic acid/thiyl radical with GSH from that of a protein thiol with GSH that had been directly oxidized by reaction with ONOO^−^. To overcome this, we generated protein sulfenic acid/thiyl radicals on the mitochondrial membranes that persisted after the added ONOO^−^ had decayed. Accordingly, we incubated mitochondrial membranes in a rapidly stirred, closed chamber with the respiratory substrate succinate (Fig. 5A). The rapid respiration by the membranes eliminated O_2_ and kept the system anaerobic (Fig. 5A), thereby extending the lifetime of any protein thiols partially oxidized to thiyl radicals or sulfenic acids. Addition of ONOO^−^ to the anaerobic system led to its complete decay after 20 s (Fig. 5B). To determine whether any partially oxidized protein thiols generated by ONOO^−^ persisted after the ONOO^−^ had decayed, we next added excess DMPO 10, 30 and 60 s after ONOO^−^ and measured DMPO-protein adduct formation (Fig. 5C). This revealed that there was still significant *N*-ethylmaleimide-sensitive DMPO-protein adduct formation even when DMPO was added 30 or 60 s after ONOO^−^ (Fig. 5C), by which time the added ONOO^−^ had decayed (Fig. 5B). To determine whether these partially oxidized protein thiols could react with GSH, we next added ONOO^−^ to an anaerobic membrane suspension and, after 30 s, when all of the ONOO^−^ would have decayed, we added [^3^H]GSH. Two minutes later, the mitochondrial membranes were isolated and processed ± dithiothreitol to quantify the amount of [^3^H]GSH bound to the membranes by a disulfide bond (Fig. 5D). This demonstrated that there was a dose-dependent increase in dithiothreitol-sensitive binding of [^3^H]GSH to membranes on exposure to ONOO^−^, which is consistent with the formation of a mixed disulfide between a partially oxidized protein thiol and GSH. Such mixed disulfides in membranes and complex I will be rapidly recycled to thiols by Grx and GSH [13,14], suggesting that this is one mechanism by which oxidized protein thiols can be recycled by the GSH pool.

## Discussion

In the present study, we have demonstrated that exposed thiols on protein surfaces are the most abundant class of thiol within the cell. The content of exposed protein thiols was significantly higher than that of the predominant low molecular thiol GSH in all fractions investigated. These findings are consistent with an important role for protein thiols in intracellular redox homeostasis [7,8]. Focussing on mitochondria, it was found that the concentration of exposed protein thiols within the mitochondrial matrix was aprpoximately 60–90 mm, which is 20–25-fold greater than that of GSH in the same compartment. Therefore, within mitochondria, the non-enzymatic reactions of thiols will be dominated by those of exposed protein thiols, and not by those of GSH.

Maintaining a high cysteine content on the surface of proteins, where the cysteine residue is not involved in any enzymatic or structural activity, is a significant cost to the organism compared to using nonsulfur amino acids [26], suggesting that surface cysteine residues may have a beneficial role. A proportion of these thiols are likely to be involved in redox regulation, and may exist in local environments that favour this. However, the proportion of exposed protein thiols in this category is likely to be small; for example, < 1% of exposed mitochondrial thiols are modified by S-nitrosation [27]. We suggest that the high concentration of exposed thiols within mitochondria plays a role in protection from nonspecific damage. This can occur because of the rapid reaction of thiols with many of the damaging species present in biological systems. Furthermore, because many of these potentially protective thiol reactions occur through the thiolate form, the higher pH in the mitochondrial matrix compared to the cytosol (7.8 versus 7.2) will make these thiols approximately five-fold more reactive than elsewhere in the cell as a result of the typical p*K*_a_ of protein thiols being approximately 8.5. The reaction rates of thiols on the surface of proteins will vary widely depending on local environment [28]. Even so, estimates of the rates of some of these potentially protective reactions can be made. The reaction of ^•^NO_2_ with the thiols of GSH or cysteine is fast (∼ 3–5 × 10^7^ m^−1^·s^−1^) [29] and the rate of reaction of ONOO^−^ with the exposed thiol of BSA is 2–3 × 10^2^ m^−1^·s^−1^ [16]. Thiols can also react with electrophiles such as the reactive aldehyde products of lipid oxidative damage [30]. For example, the rate of reaction of cysteine residues in small peptides with 4-hydroxynonenal is 1.2 m^−1^·s^−1^ [31]. Exposed protein thiols can also react with carbohydrate breakdown products such as glyoxal [32]. Although exposed protein thiols will react with H_2_O_2_, the rate is likely to be similar to that for the thiolate of cysteine (∼ 22–26 m^−1^·s^−1^) [4], which is far slower than that of H_2_O_2_ with mitochondrial PrxIII (∼ 2 × 10^7^ m^−1^·s^−1^) [33]. Similarly, the direct reaction of thiols with superoxide is possible; however, because the rate is in the range 30–1000 m^−1^·s^−1^ [4], it is negligible compared to that of manganese superoxide dismutase (MnSOD) (∼ 2 × 10^9^ m^−1^·s^−1^) [9]. Exposed protein thiols will also react very rapidly (2–4 × 10^10^ m^−1^·s^−1^) [34] with the hydroxyl radical but, because this species reacts with similarly rapidity with most other biological molecules, there will be little selectivity for the thiol. Therefore, we suggest that the high concentration of cysteine residues exposed on protein surfaces may play an important antioxidant role within mitochondria by reacting with some, but not all, damaging species within mitochondria.

**Fig. 6:**
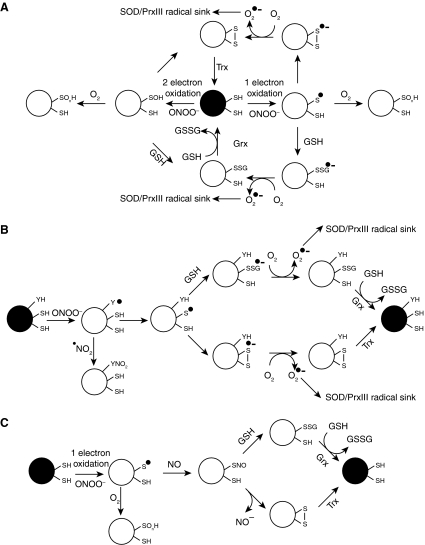
Modes of protection against oxidative damage by exposed protein thiols. The three panels show the various ways in which exposed protein thiols can protect against oxidative damage. (A) Modes of recycling of exposed protein thiols after oxidation. A schematic protein (shaded) is shown with two exposed thiols. Oxidation by ROS can generate a sulfenic acid (RSOH) or a thiyl radical. These can be irreversibly oxidized to higher thiol oxidation states (RSO_*n*_H). The sulfenic acid can be converted to an intramolecular disulfide, or form a mixed disulfide with GSH. The thiyl radical can form a radical anion intramolecular disulfide, or a mixed disulfide with GSH. These can lose an electron to O_2_ to form superoxide. The mixed disulfide thus formed can be recycled to a thiol by the action of GSH and Grx, whereas an intramolecular disulfide can be recycled by Trx. (B) Intramolecular electron transfer from a thiol to a tyrosyl radical. ROS generates a tyrosyl radical on a tyrosine residue, which is then is reduced by an adjacent thiol to generate a thiyl radical. The thiyl radical can be recycled back to a thiol by the mechanisms outlined in (A). (C) Role of NO in preventing protein oxidative damage. The thiyl radical generated by ROS can react rapidly with NO to generate a *S*-nitrosothiol. This will decrease the extent of irreversible oxidation of the thiol. The *S*-nitrosothiol can then be recycled back to a disulfide as shown.

These protective reactions of exposed protein thiols will act to block further damage, generating a modified protein thiol. In some cases, it may be acceptable to sacrifice the protein thiol; however, if this mechanism is to be effective as antioxidant process, then the oxidized protein thiols will have to be recycled. The cysteine residues along with those of methionine are the only ones that can be reversibly oxidized and reduced by biological processes. How this may occur is well established. Exposed thiols on protein surfaces will often react with ROS by one or two electron oxidation to a thiyl radical or a sulfenic acid, respectively (Fig. 6A). However, these products are unstable in the presence of O_2_, leading to further irreversible oxidation to sulfinic or sulfonic acids. To avoid this, both thiyl radicals and sulfenic acids can be rapidly recycled by reaction with other thiols. The thiyl radical will react with GSH, or with an adjacent cysteine residue, to form a disulfide radical anion, which can then react with O_2_ to form superoxide by the Winterborn reaction to regenerate a disulfide [25]. This effectively exports the radical to the mitochondrial matrix where it will be converted to H_2_O_2_ by the action of MnSOD and then degraded by PrxIII [25]. Similarly, a sulfenic acid will also react with GSH or an adjacent protein thiol to form a disulfide. These reactions with GSH generate mixed disulfides that can persist, or rapidly rearrange to form an intraprotein disulfide [14]. The intraprotein disulfides would be reduced by Trx, or by Grx and GSH, whereas the persistent mixed disulfides will be reduced by GSH catalysed by Grx [6,35–37]. The resultant GSSG or oxidized Trx will then be reduced using NADPH via TrxR or glutathione reductase. This cycle may operate in a similar way for other reversible thiol modifications such as by reactive aldehydes or carbohydrate derivatives, although it is unclear whether there are specific mechanisms to recycle all such thiol modifications. Thus, it is possible to construct an antioxidant cycle for exposed surface protein thiols that extends an earlier proposal of Thomas *et al.* [2] (Fig. 6A). The vital role of glutathione and Grx in this cycle is supported by the fact that Grx2 is essential in preventing mitochondrial oxidative damage [38,39].

In addition to being part of a general antioxidant cycle within the mitochondrial matrix, the location of the exposed thiols on the surface of proteins may also prevent oxidative damage to the proteins on which they are located. To do this, the exposed thiol will preferentially sustain the oxidative damage, rather than another amino acid, as a result of its greater reactivity with most damaging species, thereby acting as a local antioxidant on the protein surface. Accordingly, the damage to the exposed thiol will be recycled through the mechanisms outlined in Fig. 6A. This mechanism would enable oxidative damage to the protein to be continually repaired, and is similar to a proposal by which methionine residues can protect adjacent amino acid residues by being preferentially oxidized to methionine sulfoxide, which is then recycled by methionine sulfoxide reductases [40,41]. In addition to direct reaction of a surface thiol with a damaging species, it is also possible that thiols on the protein surface can funnel damage away from other amino acid residues after they have been oxidized, thereby repairing them. This is based on the work by Zhang *et al.* [42] showing that tyrosyl radicals can be reduced and repaired by an intramolecular reaction with an adjacent cysteine residue. Such an electron transfer reaction would convert the thiol to a thiyl radical, which would be recycled by the pathways outlined in Fig. 6A. This possibility is illustrated in Fig. 6B. In effect, this mechanism enables protein oxidative damage to be funnelled to a cysteine residue and then exported from the protein into the mitochondrial matrix to be dealt with by MnSOD and PrxIII. The rate of intramolecular electron transfer from the tyrosyl radical to the cysteine residue is fast (10^3^–10^4^ m^−1^·s^−1^) within simple peptides [42]. Similar reactions may occur to enable cysteine residues to reduce radicals generated on other aromatic amino acid residues such as phenylalanine and tryptophan [43], and oxidative damage could also be funnelled to methionine residues, which could then be recycled by methionine suphoxide reductases [40,41].

In the present study, we have shown that exposed protein thiols on the surface of complex I and in mitochondrial membranes decrease protein nitration by ONOO^−^. This may occur by either of the mechanisms discussed above. Thiols in the vicinity of tyrosine residues may preferentially react with the local ONOO^−^ pool. However, the rate of reaction for ONOO^−^ with protein thiols (2–3 × 10^2^ m^−1^·s^−1^) [16] is only moderate, and blocking protein thiols with *N*-ethylmaleimide had no effect on the degrdation of ONOO^−^ (Fig. 3E), suggesting that cysteine residues are unlikely to be completely effective at diverting ONOO^−^ from this reaction. Instead, we suggest that the intramolecular electron transfer mechanism shown in Fig. 6B and discussed above may explain much of the protection against tyrosine nitration by ONOO^−^ in our experiments. In this scenario, the initial formation of a tyrosyl radical by ONOO^−^ is quenched by its intramolecular reaction with a nearby thiol (Fig. 6B) before it can react further with the ^•^NO_2_ radical to form 3-nitrotyrosine. In addition, the fact that blocking thiols with *N*-ethylmaleimide accentuated the loss of complex I HAR activity on ONOO^−^ treatment also suggests that the thiols protect against damage to protein function. One further extension of the intramolecular electron transfer between tyrosyl radicals and cysteine suggested by Zhang *et al.* [42] is that it may enable the selective S-nitrosation of thiols under oxidative conditions, by generating thiyl radicals that then react with NO to give an *S*-nitrosothiol [41]. We suggest that this mechanism could also contribute to an antioxidant cycle by recycling thiyl radicals, thereby preventing their irreversible oxidation. This may occur because NO reacts rapidly with thiyl radicals (2–3 × 10^9^m^−1^·s^−1^) [42] and the *S*-nitrosothiol thus formed can be rapidly recycled back to a thiol (Fig. 6C). Thus, the formation of *S*-nitrosothiols may be part of a protein antioxidant defence cycle.

To summarize, we have shown that the quantitatively dominant thiol within cells comprises cysteine residues exposed on the surface of proteins. One reason for this may be to protect proteins from damage and we propose that exposed surface protein thiols are part of an important antioxidant cycle within mitochondria. Future work aiming to test this hypothesis should identify surface cysteine residues that do not affect the activity of a protein but which are involved in preventing oxidative damage. These findings suggest that more attention should be paid to the role of thiols exposed on the surface of proteins in the defence of cells and mitochondria against oxidative damage.

## Experimental procedures

### Preparation of tissue and mitochondrial fractions

To prepare tissue homogenates, rats were killed by cervical dislocation and the heart and liver removed to ice-cold STE (250 mm sucrose, 5 mm Tris-HCl, 1 mm EGTA, pH 7.4). The liver was homogenized using a dounce homogenizer and the heart using an Ultra-Turrax homogenizer (IKA Works, Inc., Wilmington, NC, USA) followed by dounce homogenization. The homogenates were centrifuged (3000 ***g*** for 3 min at 4 °C) giving the cell lysate as the supernatant. The cell lysate was further centrifuged (10 000 ***g*** for 10 min at 4 °C) to generate the mitochondrial fraction as the pellet, which was washed twice in STE. Portions of the supernatant from the 10 000 ***g*** centrifugation were further centrifuged (100 000 ***g*** for 15 min at 4 °C) to generate a post 100 000 ***g*** supernatant. These fractions were stored on ice for up to 3–4 h before analysis. Mitochondria for other incubations were prepared by homogenization followed by differential centrifugation in STE, or in STE containing 0.1% (w/v) fat-free BSA, for liver and heart, respectively [44], and protein contents were determined by the biuret assay. Bovine heart mitochondrial membranes were prepared by disruption of bovine heart mitochondria in a blender, followed by collection and washing by centrifugation [45]. These preparations had negligible matrix contamination, as indicated by the lack of MnSOD detected by immunoblotting, and were open fragments of mitochondrial membranes [12]. Complex I was prepared by solubilization of membranes with DDM (Anatrace Inc., Maumee, OH, USA) followed by ion-exchange chromatography [46]. Pooled fractions were further purified by gel filtration and the purified complex I was stored in 20 mm Tris-HCl (pH 7.5), 150 mm NaCl, containing 0.03% DDM, 2 mm tris(2-carboxyethyl)phosphine and 10% glycerol at −80 °C. Immediately prior to experiments, this buffer was replaced with one lacking tris(2-carboxyethyl)phosphine by centrifugation in a Micro Bio-Spin 6 (Bio-Rad, Hercules, CA, USA) chromatography column.

### Mitochondrial incubations

Mitochondria were incubated at 1–5 mg·mL^−1^ protein in KCl buffer (120 mm KCl, 10 mm Hepes, 1 mm EGTA, pH 7.4) supplemented with 5 mm succinate and 4 μg·mL^−1^ rotenone at 37 °C. To separate mitochondria into membrane and soluble fractions, mitochondria were pelleted by centrifugation (10 000 ***g*** for 5 min) resuspended in STE containing 1% DDM and centrifuged (100 000 ***g*** for 15 min at 4 °C) giving a supernatant of soluble mitochondrial proteins and a mitochondrial membrane pellet. To determine the exposed protein thiol content in the mitochondrial matrix, mitochondria were incubated with 0.1 mm of the membrane-impermeant thiol alkylating agent AMS at 30 °C for 10 min. To ensure AMS was not accessing matrix-facing protein thiols, a control experiment was carried out that measured the effect of a range of AMS concentrations on the mitochondrial matrix GSH pool. Incubation of heart or liver mitochondria with concentrations of AMS up to 0.1 mm did not deplete mitochondrial GSH, whereas AMS concentrations of 1 mm and above did and, consequently, 0.1 mm AMS was used for these experiments. Similar results to those obtained using AMS were obtained with the alternative membrane-impermeant thiol alkylating agents ([2-methylammonium)ethyl]methanio sulfonate bromide) or sodium (*S*-sulfonatopropyl)methyl thiosulfonate (both from Pierce, Rockford, IL, USA). For these experiments, the alkyating agents were incubated at 100 or 250 μm for 10 min with liver or heart mitochondria at 30 °C, and then the mitochondria were pelleted by centrifugation, lysed by freeze/thawing (×3) and the exposed protein thiols measured. For experiments with bovine heart mitochondrial membranes, the membranes were preincubated at 20 mg·mL^−1^ protein with 1 mm dithiothreitol in membrane buffer (20 mm Tris, 1 mm EDTA, pH 7.3) for 10 min at 37 °C. The membranes were then pelleted by centrifugation (10 000 ***g*** for 5 min) and washed twice in membrane buffer before use.

### Protein thiol assays

Each tissue subfraction was treated with dithiothreitol (1 mm) followed by gel filtration on a spin column (Micro Bio-Spin 6; Bio-Rad) pre-equilibrated in the appropriate buffer before measurement of exposed protein thiols in the presence of DDM (1%), or total protein thiols in the presence of SDS (2%) using the DTNB assay [47]. For this, fractions were diluted (1 : 17) with DTNB buffer (10 mm DTNB, 0.1 mm NaH_2_PO_4_, pH 8), incubated for 30 min at room temperature and *A*_412_ was measured using a plate reader (SpectraMax Plus 384; Molecular Devices, Sunnyvale, CA, USA), relative to a standard curve of 0–250 μm GSH. Protein concentration was measured by the bicinchoninic acid assay using BSA as standard [48]. Pre-treatment of tissue or mitochondrial fractions with 50 mm*N*-ethylmaleimide for 10 min prior to isolation led to loss of > 93% of exposed thiols and > 95% of total thiols.

Tissue fractions that had been incubated with 1 mm dithiothreitol, for 30 min at 30 °C, followed by dialysis (3 × 1 h, then overnight) under argon against 0.1 mm NaH_2_PO_4_ (pH 8) contained levels of exposed and total protein thiols that were similar to those obtained by centrifugal gel filtration (data not shown). The absence of dithiothreitol treatment decreased surface protein thiols in liver and heart mitochondria by 5–6%. Separating mitochondria into membrane and soluble fractions by suspending mitochondria (1 mg of protein) in 100 μL of 80 mm NaH_2_PO_4_ (pH 8) followed by freeze/thawing (×3) gave levels of exposed and total protein thiols similar to that shown in Fig. 1 (data not shown). Denaturing proteins by incubation with 8 m urea gave a total protein thiol content similar to that obtained using 2% SDS (data not shown).

### Peroxynitrite synthesis

Peroxynitrite was synthesized from sodium nitrite and acidified H_2_O_2_ followed by quenching with NaOH in a simple flow reactor as described previously [49,50]. The final solution was treated with MnO_2_ to remove excess H_2_O_2_ and then filtered and aliquots were stored at –20 °C. The concentration was determined from ε_302_ = 1670 m^−1^·cm^−1^ [51]. Control experiments were carried out using decomposed ONOO^−^, which was obtained by allowing the ONOO^−^ to decompose for 5 min before the NaOH was added.

### Electrophoresis and immunoblotting

For SDS-PAGE, samples in loading buffer were separated on 5–20% gradient gels run using a Bio-Rad Mini Protean System and then transferred to nitrocellulose. The blot was incubated with the appropriate primary antibodies followed by a secondary antibody-horseradish peroxidase conjugate and visualized by enhanced chemiluminescence (ECL Plus; GE Healthcare, Milwaukee, WI, USA). The antibodies used were mouse monoclonal raised against 3-nitrotyrosine conjugated to keyhole limpet haemocyanin (Sigma, St Louis, MO, USA); rabbit polyclonal serum raised against 5,5-dimethyl-2-(8-octanoic acid)-1-pyrroline *N*-oxide coupled to ovalbumin (Cayman Chemical Company, Ann Arbor, MI, USA); and rabbit polyclonal sera raised against the bovine complex I 75 kDa, 51 kDa or 23 kDa subunits (provided by J. E. Walker, MRC, MBU, Cambridge, UK).

BN-PAGE was used to isolate mitochondrial complex I from mitochondrial membranes [23]. Pelleted mitochondrial membranes (0.5 mg of protein) were resuspended in 60 μL of extraction buffer [1% DDM, 0.75 mε-amino-*n*-caproic acid (ACA), 50 mm Bis-Tris-HCl, pH 7.0] and incubated on ice for 15 min and then clarified by centrifugation in an Airfuge™ (Beckman Coulter, Fullerton, CA, USA) at 17 psi (∼ 100 000 ***g***) for 15 min. To 50 μL of supernatant, 3.75 μL of BN gel loading buffer [0.5 m ACA, 5% (w/v) Serva Blue G250] was added, and then 20 μL of sample was resolved on a 1 mm thick 5–12% acrylamide gradient gel containing 0–0.2% (w/v) glycerol, 1.5 m ACA, 150 mm Bis-Tris-HCl pH 7.0 (4 °C), overlaid with a 3.9% acrylamide stacking gel in the same buffer. The anode buffer was 50 mm Bis-Tris (pH 7.0) and the cathode buffer was 0.02% (w/v) Coomassie blue G250, 50 mm tricine, 15 mm Bis-Tris (pH 7.0). The gel was run at 4 °C for 1 h at 100 V and then overnight at 40 V in cathode buffer without coomassie blue. Bands corresponding to complex I were excised and incubated in denaturing alkylating buffer (2% SDS, 50 mm*N*-ethylmaleimide, 125 mm Tris, pH 7.0) at room temperature for 5 min and then, resolved on a 12% SDS-PAGE gel, transferred to nitrocellulose using a Bio-Rad Trans-Blot Semi-Dry transfer cell and then probed with antibodies. For re-probing, blots were incubated in stripping buffer [62.5 mm Tris-HCl, 2% (w/v) SDS, 100 mm 2-mercaptoethanol, pH 6.8] for 30 min at 50 °C with constant agitation, washed (×5) with PBST [137 mm NaCl, 10.2 mm NaHPO_4_, 2.7 mm KCl, 1.8 mm KH_2_PO_4_, 0.05% (v/v) Tween 20, pH 7.4] before blocking the membrane and repeating the immunoblotting procedure with a different antibody.

### Other assays

Complex I activity was assessed as the NADH:HAR activity measured by the decrease in *A*_340_ [24]. Accordingly, 1.5 mL of 125 μm NADH and 1 mm HAR in 50 mm KCl, 10 mm Tris, 1 mm EDTA (pH 7.4) was equilibrated at 30 °C with stirring in an Aminco DW2000 spectrophotometer (Aminco International Inc., Lake Forest, CA, USA). The reaction was started by addition of mitochondrial membranes (20 μg of protein) or isolated complex I (1 μg of protein). To measure GSH, protein was precipitated by the addition of 5% sulfosalicylic acid followed by centrifugation (10 000 ***g*** for 5 min) and the supernatants taken for measurement of GSH and GSSG using the recycling assay [11]. Glutathione protein mixed disulfides were determined by reducing the protein pellet with sodium borohydride and measuring the released GSH using the glutathione recycling assay [11]. To measure the binding of [^3^H]GSH to mitochondrial membranes, membranes (1 mg·mL^−1^ protein) were incubated in membrane buffer at room temperature (∼ 23 °C) in the stirred 3 mL chamber of an oxygen electrode (Rank Brothers, Bottisham, UK) in the presence of 2 mm succinate and 4 μg·mL^−1^ rotenone. [^3^H]GSH (100 μm, 19 246 Bq·mmol^−1^, 37 MBq·mL^−1^; American Radiolabeled Chemicals Inc., St Louis, MO, USA) was diluted 1 : 1 with 20 mm unlabelled GSH to make a 10 mm [^3^H]GSH stock solution. To assess binding of [^3^H]GSH to the membranes, the membrane suspension was divided in two and one half was incubated with 1 mm dithiothreitol and the other with carrier for 2 min. Next, the membranes were pelleted by centrifugation (10 000 ***g*** for 5 min), the protein pellets dissolved in 50 μL of 20% Triton X-100 and suspended in 3 mL of Fluoran-Safe 2 scintillant and the [^3^H]GSH content measured using a Tri-Carb 2 800 TR Perkin Elmer scintillation counter (Perkin Elmer, Boston, MA, USA) with appropriate quench correction. Samples of the [^3^H] GSH stock solution were measured to determine its specific activity.

## Abbreviations

ACA: ε-amino-*n*-caproic acid
AMS: 4-acetamido-4′-maleimidylstilbene-2,2′-disulfonic acid
BN-PAGE: blue native gel-PAGE
DDM: *n*-dodecyl-β-d-maltopyranoside
DMPO: 5,5-dimethyl-1-pyrroline-*N*-oxide
DTNB: 5,5′-dithiobis(2-nitrobenzoic acid)
Grx: glutaredoxin
GSH: glutathione
GSSG: glutathione disulfide
HAR: hexa-ammineruthenium (III) chloride
MnSOD: manganese superoxide dismutase
ONOO^−^: peroxynitrite
Prx: peroxiredoxin
ROS: reactive oxygen species
tBHP: *tert*-butyl hydrogen peroxide
Trx: thioredoxin
TrxR: thioredoxin reductase
